# Theoretical Study of Retinol, Niacinamide and Glycolic Acid with Halloysite Clay Mineral as Active Ingredients for Topical Skin Care Formulations

**DOI:** 10.3390/molecules26154392

**Published:** 2021-07-21

**Authors:** Ana Borrego-Sánchez, Claro Ignacio Sainz-Díaz, Luana Perioli, César Viseras

**Affiliations:** 1 Center for Human Technologies, Italian Institute of Technology, Via Enrico Melen 83, 16152 Genoa, Italy; 2 Department of Pharmacy and Pharmaceutical Technology, Faculty of Pharmacy, Campus de Cartuja s/n, University of Granada, 18071 Granada, Spain; 3 Instituto Andaluz de Ciencias de la Tierra (Consejo Superior de Investigaciones Científicas-University of Granada), Av. de las Palmeras 4, Armilla, 18100 Granada, Spain; ignacio.sainz@iact.ugr-csic.es; 4 Department of Pharmaceutical Sciences, University of Perugia, Via del Liceo 1, 06123 Perugia, Italy; luana.perioli@unipg.it

**Keywords:** halloysite, retinol, niacinamide, glycolic acid, adsorption, molecular modeling

## Abstract

The adsorption of retinol, niacinamide and glycolic acid active ingredients on the internal surface of halloysite in an aqueous environment was explored at the molecular level by means of calculations based on quantum mechanics and force fields from empirical interatomic potentials. These active ingredients are stably adsorbed on the internal surface of halloysite forming hydrogen bonds between the hydrogen, oxygen and nitrogen atoms with the hydroxyl groups of the inner surface of the halloysite. In addition, electrostatic interaction between these active ingredients with the water molecules was observed. Therefore, the theoretical results indicate that the adsorption of these active principles is favourable in the halloysite nanotube, which allows directing future experimental investigations for the development and design of retinol, niacinamide and glycolic acid with halloysite nanotubes systems, which may be topical formulations for skincare.

## 1. Introduction

Natural, modified, or synthetic clay minerals are some of the most used ingredients in the pharmaceutical area. The interest in their numerous applications has been increasing in the field of drug and active ingredient delivery systems. This is because clays have a wide variety of properties that make them advantageous and useful ingredients [1,2]. Specifically, in cosmetics, clays have been used as active ingredients and as excipients, with the main purpose of improving the cosmetic formulation. For example, they have been used as viscosifiers [3,4], gelling and emulsifying agents in the formation of emulsions [5], stabilizing agents for suspensions and agents that confer thixotropic properties to suspensions [6], and moisturizers in the preparation of cosmetics for the skin and hair [5]. Clay minerals are also used as binders in the formulation of toothpastes [7], and as sunscreens because they reflect ultraviolet radiation and serve as support for other UV filters [8]. Other uses are as powders, in the formulation of deodorant creams, in dry anti-grease shampoos and in facial cosmetic formulations, as a base for make-up, compact powders and hydrating masks [9]. Additionally, currently, they act as active ingredients in dermocosmetic products for topical application, because clays have different properties for the maintenance and care of the skin, including the delay in aging, wound healing, anti-inflammatory, soothing of irritations, exfoliants, actiacne and improving the skin appearance and texture [10,11].

In particular, halloysite is a nanotubular clay of the kaolin group, its chemical composition is the same as kaolinite (Al_2_Si_2_O_5_(OH)_4_·nH_2_O), with a dioctahedral 1:1 structure. Generally, the length of the nanotubes is 0.2–1.5 µm, and the inner and outer diameters are in the ranges of 10–30 nm and 40–70 nm, respectively [12]. The inner surface is aluminum hydroxide (with positive charge) and the external surface is silicon dioxide (with negative charge). The halloysite structure and composition provide properties, such as high-absorbent, biocompatible and low cost, that make them versatile materials for pharmaceutical applications, as other clay minerals [13,14,15,16,17]. In the cosmetic field, halloysite has been studied as a skin cleanser agent [18]. Recently, halloysite nanotubes have been used to encapsulate hair dyes, allowing the use of different low water-soluble compounds, and hair coating has also been observed through physical adsorption and self-assembly of the nanotubes [19,20]. In addition, halloysite with titanium oxide as sunscreen [21], and hybrid halloysite nanotubes and keratin have been studied, producing the coating of the capillary surface and thus protecting it against ultraviolet radiation [22]. Within the field of cosmetics, most of the products used nowadays, including those that contain halloysite or other clays, are intended for topical application, specifically, the formulation of anti-aging cosmetic products that delay the appearance of these signs, as well as other skincare maintenance products that are booming [23,24].

Apart from clays, there are other ingredients widely used in this type of dermocosmetic formulations and that can be used topically incorporated into halloysite, seeking many positive effects in the same formulation. Pure vitamin A, also called retinol, has several roles depending on the concentration at which it is used in the cosmetic formulation. At low concentrations, it is a powerful antioxidant and neutralizer of free radicals, and it has skin-softening effects by improving the synthesis of collagen and elastin, which contributes to the hydration and luminosity of the skin. At higher concentrations, retinol has keratolytic activity, decreasing the stratum corneum (cell renewal), improving wrinkles and acne marks, and having depigmenting properties. Hence, retinol is frequently used as an active ingredient in many skincare formulations to reduce photoaged and aged skin, wrinkles, hyperpigmentation, acne, psoriasis, among others [25,26]. However, it has some disadvantages in its formulations due to its low aqueous solubility and photosensitivity (highly sensitive to light and oxygen). Additionally, retinol has a narrow therapeutic window, leading it to be ineffective at low concentrations and toxic at high concentrations [27]. Its administration as a hybrid halloysite–retinol system could show good results, improving the elaboration of the formulations and their efficacy. Clay minerals could improve their aqueous solubility, as has been shown in their interaction with other drugs [28]. Moreover, clays could protect retinol from sunlight (physical sunscreen clays). Thus, the topical effects of clays would act synergistically with retinol.

Niacinamide is also among the top ten most used antioxidants in recent years [24]. Niacinamide, also known as nicotinamide, is a form of vitamin B3 and it is present in human cells as a precursor of nicotinamide adenine dinucleotide. The topical administration of this cosmetic ingredient has shown benefits across a wide range of skin conditions. Niacinamide has been proposed as an antiaging cosmetic for its antioxidant effects. It has also shown benefits in inflammatory, acneic, hyperpigmentary and photoprotection processes, and it decreases cutaneous pigmentation, and thus its efficacy in the treatment of rosacea, acne and autoimmune blistering disorders such as bullous pemphigoid has been reported [29,30,31,32].

In addition, another compound currently relevant in skincare cosmetics is glycolic acid or hydroxyacetic acid which is the shortest chain alpha hydroxy acid. This active ingredient has an action on the deepest surface of the skin and after its facial application produces superficial chemical exfoliation, being one of the most used peels together with retinoids [33]. Hence, glycolic acid is widely indicated to rejuvenate the skin overall because when it is applied, the skin surface is exfoliated in order to renew the skin [34]. This active ingredient is used for moderate photodamage, actinic damage, acne, seborrheic skin, rosacea and pigmentary disorders [35]. Therefore, glycolic acid applications are intended to reduce wrinkles, stretch marks, scars, acne, lack of luminosity or skin damaged by the sun [36,37].

For these reasons, the formulation of halloysite with these active ingredients (retinol, niacinamide and glycolic acid) would bring great advantages in the cosmetic field. Halloysite would be complemented with these ingredients acting as an active ingredient as well as an excipient serving as a vehicle and protecting active ingredients inside the nanotube from degradation. In the last few decades, calculations at the atomic and molecular levels have shown to be useful tools for design drugs and pharmaceutical composites in order to explore potential applications. Specifically, the adsorption of organic molecules on halloysite has been previously studied with molecular modeling [38,39,40,41]. In this work, the halloysite–retinol, halloysite–niacinamide and halloysite–glycolic acid systems are studied with computational methods as the first step in the development of cosmetic skincare formulations.

## 2. Methodology

### 2.1. Models

The retinol, niacinamide and glycolic acid crystals were taken from the crystallographic data of CCDC num. 1,284,683 [42], 817,410 [43], 1,011,488 [44], respectively. Subsequently, the molecules were extracted and modified from the crystals in order to study their adsorption in halloysite nanotube. For it, a 1 × 1 × 2 supercell of halloysite with the formula Al_152_Si_152_O_380_(OH)_304_ and with 1292 atoms was generated. The halloysite supercell was created from the unit cell of the nanotube that was described in previous works [45,46,47], which has an internal layer of aluminium hydroxide octahedral, with an internal diameter of 27 Å, joined to an external layer of silicon oxide tetrahedra. This small halloysite model was chosen in a balance between the computational effort and a representative description of the mineral surface in the confined space of the clay mineral. The adsorption complexes created, halloysite–retinol, halloysite–niacinamide and halloysite–glycolic acid, were good models to reproduce the interactions between the active ingredients at the molecular level.

### 2.2. Molecular Modeling Methodology

The optimization of the unit cell of halloysite nanotube structure was performed with quantum mechanical calculations by using Density Functional Theory (DFT) with CASTEP code of the Materials Studio package [48] the Perdew–Burke–Ernzerhof (PBE) correlation exchange parameterization. On-the-fly generated (OTFG) ultrasoft pseudopotentials were used with Koelling–Harmon relativistic treatment [49], and the cut-off energy of the calculation was 300 eV [48]. After the optimization of the halloysite unit cell, the 1 × 1 × 2 supercell was created to study the adsorption of the retinol, niacinamide and glycolic acid molecules.

These molecules were optimized with the Compass force field (FF) by using the Forcite program that has provided good results in previous studies [48,50]. For non-bonding interactions, the Coulomb and van de Waals interactions were calculated by the Ewald and atom-based methods, respectively, with a cut-off of 18.5 Å. The water geometry was optimized using the same methodology.

Different conformations and orientations between the retinol, niacinamide and glycolic acid inside the halloysite nanotube were randomly explored using Monte Carlo methods with the Compass FF and the Adsorption Locator module [48]. The more stable active ingredient-clay complexes were selected. Later, the selected adsorption models were filled with the optimized water molecules with 1 g/cm^3^ density using the Compass FF and Amorphous Cell module [48]. In these complexes, the DFT optimized structure of the halloysite was fixed except for the hydrogen atoms of the inner surface. Subsequently, the complexes with the water molecules were reoptimized using again Compass FF and Forcite module with a cut-off of 18.5 Å.

The adsorption energies of these complexes were compared according to the equation:

Δ*E*_ads_ = (*E*_complex_) − (*E*_active_ + *E*_clay_). (1)

For this, the energies of each optimized active-clay adsorption complex with water, *E*_complex_, the optimized active ingredients, *E*_active_, and the optimized halloysite nanotube with water, *E*_clay_, was calculated using the Compass FF and the Forcite program with a cut-off of 18.5 Å [48].

## 3. Results

**Figure 1:**
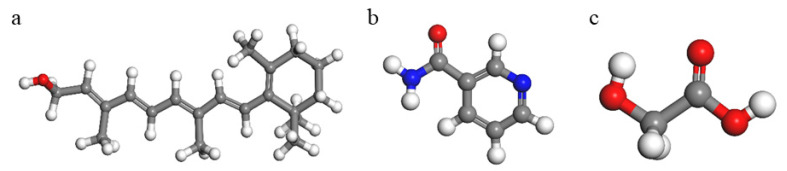
Optimized molecular structures of retinol (**a**), niacinamide (**b**) and glycolic acid (**c**).

Firstly, the geometry of retinol, niacinamide and glycolic acid molecules was optimized with Compass FF as described above. In Figure 1, the optimized structures are shown. In addition to the bioactivity of these drugs, the chosen molecules represent a wide range of molecular properties, one with a long hydrophobic chain as retinol, one more polar with an aromatic ring and amide group as niacinamide, and another more polar and small molecule with reactive functional groups as the glycolic acid. In the optimized molecule of niacinamide, the carbonyl group and amide N atom are co-planar with the aromatic ring. In the same way, the hydroxyl and carbonyl groups of glycolic acid are co-planar with hydroxyl groups in a syn conformation, oriented towards the carbonyl group.

Subsequently, the unit cell of the halloysite nanotube was optimized with the DFT method as described above, and then the supercell 1 × 1 × 2 was created. The adsorption of the optimized retinol molecule in the inner of the halloysite nanotube was explored. Monte Carlo methods using the Compass FF were performed to sample the large conformation space of these macromolecular systems and to determine the most stable active ingredients–halloysite complexes. From these simulations, the 10 structures with the lowest energy were compared. In all cases, the molecules were adsorbed in the internal surface of the halloysite with a parallel arrangement and similar orientation between the clay surface and the drug with no large variance were found. Therefore, the lowest-energy structure was taken as representative of this sampling. Other orientations are not expected to have a significant role due to their higher relative energy.

**Figure 2:**
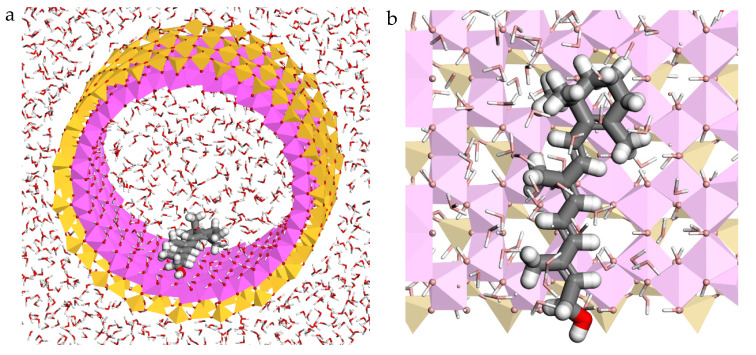
Optimized adsorption complex of the retinol adsorbed on the internal surface of halloysite nanotube with waters, views from (001) (**a**) and (100) (**b**) planes. The silicon, aluminium, hydrogen, carbon, and oxygen atoms are presented in yellow, pink, white, grey, and red respectively.

The retinol–halloysite complex was selected, filled with water molecules and reoptimized again with Compass FF. The results showed that the retinol inside the halloysite was oriented in a perpendicular direction with respect to the *c* axis of the nanotube and in parallel with respect to the mineral surface (Figure 2). The most important interaction between the active molecule and the mineral is a strong hydrogen bond between the hydroxyl O atom of retinol and the H atoms of the OH groups of the inner surface of halloysite with d(O_RET_…H_HAL_) = 1.65 Å. In addition, electrostatic interactions were found, between the H atoms of the retinol and the O atoms of the clay mineral inner surface with d (H_RET_…O_HAL_) about 2.54–2.76 Å. The water molecules that surround retinol also present electrostatic interactions with this molecule with d(H_RET_…O_H2O_) = 2.61–2.84 Å (Figure 2). The adsorption energy of this complex was −57.52 kcal/mol. Therefore, the adsorption of the retinol on the internal surface of the halloysite is exothermic and favourable.

**Figure 3:**
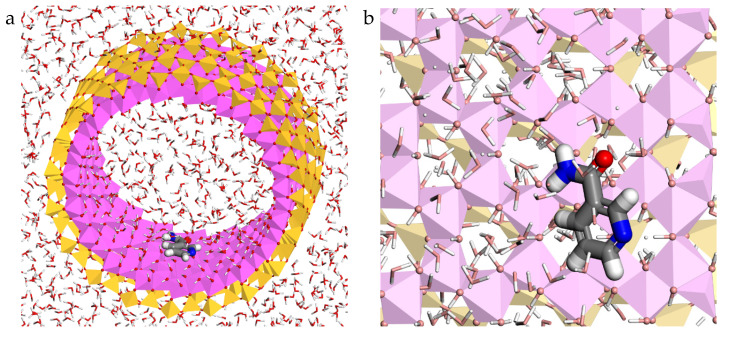
Optimized adsorption complex of the niacinamide adsorbed on the halloysite internal surface with water molecules, views from (001) (**a**) and (100) (**b**) planes. The Si, Al, H, C, O, and N atoms are represented in yellow, pink, white, grey, red, and blue colours, respectively.

The most stable complex of niacinamide adsorbed on the internal surface of the halloysite was also found with Monte Carlo methods using the Compass FF. This complex was selected, filled with water molecules and reoptimized again with Compass FF (Figure 3). The results indicated that the niacinamide was oriented in a perpendicular direction with respect to the c axis of the nanotube and in parallel with respect to the halloysite surface. Therefore, there are strong hydrogen bonds between the oxygen and the nitrogen atoms of niacinamide with the H atoms of the OH groups of the inner surface of halloysite with d(O_NIAC_…H_HAL_) = 1.79 Å and d(N_NIAC_…H_HAL_) = 1.87 Å, respectively. Electrostatic interactions were found between the H atoms of niacinamide and the O atoms of halloysite with d(H_NIAC_…O_HAL_) about 2.61–2.85 Å. Additionally, hydrogen bonds between the oxygen, nitrogen and hydrogen atoms of niacinamide with the water molecules around d(O_NIAC_…H_H2O_) = 1.81 Å, d(N_NIAC_…H_H2O_) = 1.88 Å, and d(H_NIAC_…O_H2O_) = 2.28 Å, respectively were observed (Figure 3). In addition, the niacinamide–halloysite complex adsorption energy was −45.45 kcal/mol, hence the adsorption of this active ingredient on the halloysite confined surface is a favourable process.

**Figure 4:**
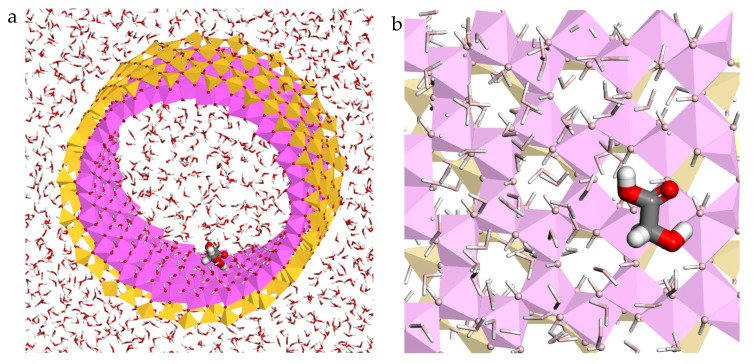
Optimized adsorption complex of the glycolic acid adsorbed on the nanotube inner surface in an aqueous environment, views from (001) (**a**) and (100) (**b**) planes. The Si, Al, H, C, O, and N atoms are represented in yellow, pink, white, grey, red, and blue colours, respectively.

Finally, the adsorption of the glycolic acid on the inner surface of the halloysite was also studied initially with Monte Carlo methods using the Compass FF. After that, the most stable complex of glycolic acid–halloysite was selected, filled with waters and reoptimized again with the same FF. With this active ingredient, the optimized adsorption complex showed that the glycolic acid was also placed in a perpendicular direction with respect to the c axis of the nanotube and in parallel with respect to the clay mineral (Figure 4). The main interactions between glycolic acid and the halloysite structure were showed, specifically between the hydrogen and oxygen atoms of glycolic acid with the oxygen and hydrogen atoms of the mineral internal surface with d(H_AC_…O_HAL_) = 1.65–2.56 Å and d(O_AC_…H_HAL_) = 2.11–2.68 Å, respectively. In addition, interactions between the hydrogen and oxygen atoms of glycolic acid with the oxygen and hydrogen atoms of the water molecules were found with d(H_AC_…O_H2O_) = 1.68 Å and d(O_AC_…H_H2O_) = 2.19 Å, respectively (Figure 4). The adsorption energy of this complex was calculated with the equation described above. The results showed adsorption energy of −21.22 kcal/mol, therefore the adsorption of the glycolic acid on the internal surface of the halloysite is also favourable. Nevertheless, this energy is lower than in the above drugs.

In summary, with the results obtained with the Monte Carlo simulations, it was observed that the adsorption of these molecules (retinol, niacinamide and glycolic acid) is more favorable in the internal surface of the halloysite. Specifically, the molecules are positioned parallel to the surface and in a perpendicular position with respect to the c axis of the nanotube. The geometry optimizations of the adsorption complexes in a water environment showed the main hydrogen bonds and electrostatic interactions between the drug, the surface of the halloysite and the water molecules; as well as that retinol, niacinamide and glycolic acid are stably adsorbed in the clay mineral nanotube.

Considering these findings in the context of possible applications in the field of dermocosmetics, it can be stated that the three active ingredient–clay complexes have favourable affinities and the adsorption physicochemical properties suggest the continuation of studies focused on preparing cosmetic formulations based on these systems. Note that the retinol–halloysite hybrid material showed the best performance among the three studied here and would be then the optimal one for this purpose.

## 4. Conclusions

Retinol, niacinamide and glycolic acid are molecules whose use is currently booming, due to the fact that they have a great variety of beneficial effects on the skin, like halloysite. The combined use of these aforementioned active ingredients with halloysite clay minerals may lead to formulations for skincare.

In this work, the adsorption of these active ingredients (retinol, niacinamide and glycolic acid) on the halloysite nanotube was studied with theoretical calculations allowing to investigate these adsorption phenomena at the atomistic level, as the first stage in the design and development of these active ingredient–halloysite formulations. The results showed that the main interactions between these active ingredients and the halloysite in an aqueous environment are hydrogen bonds or electrostatic interactions between mainly oxygen and hydrogen atoms of the active ingredients with hydrogen or oxygen atoms of the internal surface of the halloysite nanotube. Furthermore, retinol, niacinamide and glycolic acid were adsorbed inside the nanotube with an exothermic process, and the adsorption of these active ingredients on the confined halloysite surface was favourable. This indicates that retinol, niacinamide and glycolic acid could be experimentally adsorbed on the halloysite to obtain topical formulations for skincare.

## Data Availability

Not applicable.

## Sample Availability

Samples of the compounds are not available from the authors.
